# Better early outcome with enhanced recovery total hip arthroplasty (ERAS-THA) versus conventional setup in randomized clinical trial (RCT)

**DOI:** 10.1007/s00402-023-05002-w

**Published:** 2023-08-08

**Authors:** Julia Götz, Günther Maderbacher, Franziska Leiss, Florian Zeman, Matthias Meyer, Jan Reinhard, Joachim Grifka, Felix Greimel

**Affiliations:** 1 Department of Orthopedics, University Medical Center Regensburg, Asklepios Klinikum Bad Abbach, Kaiser-Karl-V.-Allee 3, 93077 Bad Abbach, Germany; 2 Center for Clinical Studies, University Medical Center Regensburg, Franz-Josef-Strauss-Allee 11, 93053 Regensburg, Germany

**Keywords:** Enhanced recovery after surgery (ERAS), Total hip arthroplasty (THA), Randomized clinical trial (RCT), Timed up and go test (TUG)

## Abstract

**Introduction:**

Numbers of total hip arthroplasty (THA) are steadily rising and patients expect faster mobility without pain postoperatively. The aim of enhanced recovery after Surgery (ERAS) programs in a multidisciplinary setup was to keep pace with the needs of quality and quantity of surgical THA-interventions and patients’ expectations.

**Methods:**

194 patients undergoing THA procedures were investigated after single-blinded randomization to ERAS (98) or conventional setup group (96). Primary outcome variable was mobilization measured with the Timed Up and Go Test (TUG) in seconds. Secondary outcome variables were floor count and walking distance in meters as well as rest, mobilization and night pain on a numerous rating scale (NRS). All variables were recorded preoperatively and daily until the sixth postoperative day. To assess and compare clinical outcome and patient satisfaction, the PPP33-Score and PROMs were used.

**Results:**

No complications such as thromboembolic complications, fractures or revisions were recorded within the first week postoperatively in either study group. Compared to the conventional group, the ERAS group showed significantly better TUG (*p* < 0.050) and walking distance results after surgery up to the sixth, and floor count up to the third postoperative day. On the first and second postoperative day, ERAS patients showed superior results (*p* < 0.001) in all independent activity subitems. Regarding the evaluation of pain (NRS), PPP33 and PROMS, no significant difference was shown (*p* > 0.050).

**Conclusion:**

This prospective single-blinded randomized controlled clinical trial was able to demonstrate excellent outcome with comparable pain after ERAS THA versus a conventional setup. Therefore, ERAS could be used in daily clinical practice.

## Introduction

In the 1990s, Enhanced Recovery after Surgery (ERAS) was first described in general surgery by the Danish surgeon H. Kehlet [1]. At the same time, Bardram et al. reported results in colorectal surgery showing the effectiveness of multimodal approaches with sufficient pain management considering early mobilization with associated complication reduction and shorter hospital stay [2]. In the future, a significant increase in numbers of total hip arthroplasty (THA) is expected [3]. Consequently, attempts have been made to increase efficacy regarding the preoperative, perioperative and postoperative THA treatment pathway to expedite patient convalescence [4]. A large meta-analysis with a total of 9936 cases was able to show that postoperative length of hospital stay was significantly lower (*p* < 0.01) and fewer complications occurred in the ERAS group than in the non-ERAS control group (*p* = 0.03) [5]. The previous ERAS literature had focused on hospital duration and readmission [6–8], but subjective satisfaction and mobility, which so far have not been evaluated into depth, play an increasingly important role. To accelerate the recovery process, it is important to reduce postoperative physical and psychological stress, thereby decreasing recovery time [9, 10]. In this context, pain management plays a major role. The aim of pain management is to improve patient’s satisfaction and functional outcome after THA [11]. Developments of multimodal analgesia seems to be more and more important in the perioperative and postoperative care of patients undergoing ERAS THA [12, 13]. For this reason, the ERAS concept includes regional anesthetic techniques in combination with an opioid-sparing multimodal analgesic approach as well as preoperative patient education, minimally invasive surgery technique and early mobilization [14]. Based on results and reports in the current literature [15, 16], we established a standardized ERAS THA treatment protocol in our department.

**Fig. 1:**
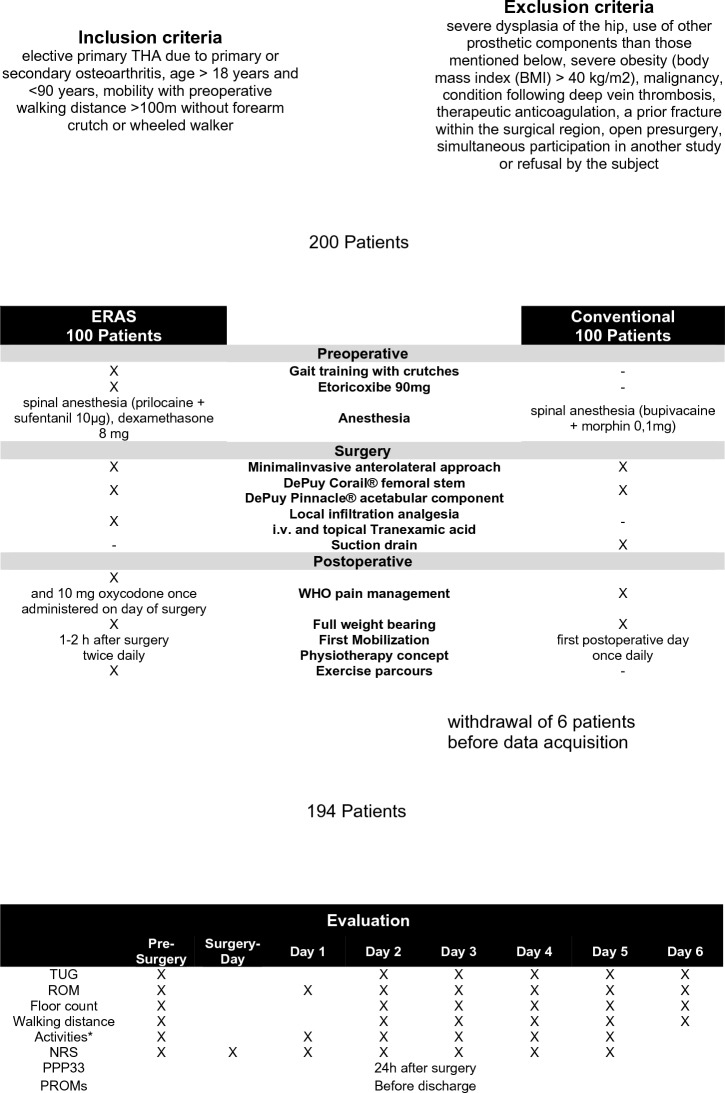
Flowchart: Study group enrollment. Activities are: Getting up/Personal hygiene/Going to the toilet/Dressing/Sitting and Walking. The postoperative days are abbreviated as Day 1/Day 2 and so on

Most published studies did not emphasize on pain, subjective outcome and mobility. Moreover, no scientific high-quality study in a controlled study design was published. We hypothesized that ERAS patients would reach autonomous mobility significantly faster while having a comparable pain level. Therefore, this is the single-blinded prospective randomized controlled clinical study (RCT) to assess mobilization and mobility with the widespread used “time up and go test” (TUG) [17] after ERAS THA versus a conventional setup. In addition, pain level, subjective scores, mobilization and PROMS of the patients were evaluated after surgery (Fig. 1).

## Methods

In this single-blinded prospective randomized controlled study, 200 patients who underwent primary THA between mid of 2019 until end of 2021 in a single center were included.

### Blinding

All included patients were randomized by an independent person. A block randomization with four blocks of 50 patients was performed, grouped by the 2 pathways: ERAS and conventional setup group. Randomized group allocation was done by envelope. Only the surgeon knew which group the patient was assigned to (single blinded), this had regulatory reasons (radiation and pharmaceutical regulations in Germany for the use of X-rays and local infiltration analgesia). In addition, the ERAS group and the conventional setup group were treated in different wards at different floors to eliminate contact and exchange.

### Trial design and participants

Inclusion criteria were: elective primary THA due to primary or secondary osteoarthritis. Exclusion criteria were: age < 18 years or > 90 years, severe dysplasia of the hip, use of other prosthetic components than those mentioned below, severe obesity (body mass index (BMI) > 40 kg/m2), malignancy, condition following deep vein thrombosis, therapeutic anticoagulation, immobility with preoperative walking distance < 100 m with forearm crutch or wheeled walker, a prior fracture within the surgical region, open pre-surgery, simultaneous participation in another study or refusal by the subject. The study was approved by the local Ethics Committee (approval number 19-1308-101). The study was applied in accordance with the ethical standards of the Declaration of Helsinki 1975. No changes after trial commencement were made with regard to course of the study. 6 patients withdrew approval after randomization but before data acquisition with the justification, that the extent of data evaluation is too extensive. No patient died within the study period. 194 patients were finally evaluated. The flowchart (Fig. 1) shows the enrollment, differences and evaluation of the study group.

### Interventions

All ERAS patients received preoperative multidisciplinary lecture and gait training with crutches. Furthermore, Etoricoxibe 90 mg was applied once an hour before surgery as preemptive analgesia. The ERAS group underwent surgery in spinal anesthesia (prilocaine 1% hyperbaric 4 ml = 80 mg and sufentanil 10 μg as standard) with intravenous administration of dexamethasone (8 mg) [42]. In contrast, the control group received a long-acting spinal anesthesia (bupivacaine 0.5% = 4 ml and 0.1 mg morphine).

A minimally invasive anterolateral approach, and a cementless collarless THA (DePuy Corail^®^ femoral stem, DePuy Pinnacle^®^ acetabular component, DePuy Orthopaedics, Warsaw, IN, USA) were used in all cases in both groups. In contrast to conventional THA, the ERAS group received both periarticular and subcutaneous infiltration analgesia (2 × 50 ml 0.2% ropivacaine, for deep infiltration with 0.5 mg epinephrine). Furthermore 1 g tranexamic acid was topically administered after preparation and another 2 g were applied intraarticularly through the closed fascia. Another difference in the ERAS group was the omission of suction drains, the application of wound adhesive after wound closure and the use of a transparent wound dressing. In both groups, full weight bearing was allowed and range of motion was not restricted. In the ERAS group, mobilization began as soon as peripheral sensory and motor function were obtained, usually 1–2 h after surgery. After stimulating cardiovascular and thrombosis prophylaxis exercises, the first walking exercises with crutches were performed under physiotherapeutic supervision. The aim for the day of surgery was a walking distance of at least 50 m. In contrast, mobilization after conventional THA started from the first postoperative day after the prolonged sensory and motor function restrictions had relieved. The suction drains were removed on the second postoperative day. Subsequently, standardized physiotherapy concept was performed twice-daily in the ERAS group and once-daily in the conventional group. Physical therapy included mobilization, muscle strengthening, thrombosis and pneumonia prevention. From postoperative day 1, a parcours was used for the ERAS group patients to increase the intensity of movement and training; patients received additional physiotherapy twice a day. The ERAS exercise parcours consists of gait training, various muscle strengthening exercises, and instructions to improve coordination. Furthermore, a mirror wall with a support bar on the ward was regularly used at the ERAS ward. Here, patients could repeat the educated exercises several times a day independently and under self-monitoring to reflect on their gait pattern, and to self-correct possible errors. After surgery, 10 mg oxycodone was administered once on the day of surgery within the ERAS group. In our department, a standardized pain management concept was established regarding the recommendations within the World Health Organization (WHO) analgesic ladder [18] composed of the following steps in both study groups: Oral pain medication consists of ibuprofen (600 mg) administered three times daily and metamizole (500 mg) regularly four times daily. Depending on NRS values, patients can receive tramadol 100 mg (40 drops) or oxycodone 10 mg as optional additional analgesics “rescue medication”, if needed. In the recovery ward, 3 mg of piritramide was optionally administered as needed, depending on the numerical rating scale (NRS; 0 = no pain; 10 = worst pain imaginable). To ensure the blinded study design and standardized comparability, all patients were hospitalized for 1 week after surgery, which is usual in inpatient care after THA in Germany.

Our hypothesis was that ERAS patients would become mobile significantly faster while having a comparable pain level were measured with the primary outcome variable TUG-score and the secondary outcome variable pain on a numeric rating scale (NRS). In addition, subjective scores, daily mobilization and PROMS of the patients were evaluated after surgery (Fig. 1).

### Activity

The widespread and standardized “Timed Up and Go Test” (TUG) [19] was carried out preoperatively and daily starting from the second to the sixth postoperative day by an independent physiotherapist. All study patients performed the test once, if a clear error was made, they were asked to repeat it. The study patient sat in a sturdy armchair with a back which was placed at the end of a 3-m marked walking path. First general instructions about the task, including walking at a normal rather than a rapid speed was given. The time measurement included the time from standing up out of the chair, walk three meters, turn around, walk back to the chair and sit down. Before starting, the examiner checked that the patients were seated in the chair with their back against the backrest and the arms resting on the armrests.

The patient’s independent activities were evaluated preoperatively and daily starting from the first to the fifth postoperative day. The recorded standard activities were divided into getting up, personal hygiene, dressing, sitting, walking and going to the toilet (not performed/performed with much help/performed with little help/performed without help). In addition, the passive range of motion (flexion/extension) was measured in degrees, the walking distance in meters and the floor count in absolute floors. All of these variables made it possible to evaluate detailed information of mobilization in the immediate postoperative course during the first postoperative week.

### Pain, PPP33 and PROMs

To assess satisfaction, pain and health related quality of life a subjective questionnaire was used. Rest, mobilization and night pain were recorded preoperatively and daily starting on the day of surgery up to day 5. Pain was assessed using the numerical rating scale (NRS) from zero (= no pain at all) to ten (= worst pain ever possible). To assess the perioperative setting and the patient satisfaction, the PPP33 (Patient assessment in the perioperative phase) was used on the second postoperative day [20]. The questionnaire contains 33 questions which can be answered on a 4-point scale (Strongly disagree, disagree, agree, strongly agree). The subgroups with different weightings in the evaluation were information (seven items), patient autonomy (six items), communication (six items), physical discomfort (five items), pain (three items), rest/recovery (two items), anxiety (two items), and clinic performance (two items). The overall mean score for the PPP33 is ranged from 0 to 100. Generally, a higher PPP33 score represents a better outcome. The following non-validated PROMs were analyzed 1 week after surgery: Was the operation successful in your eyes (yes/no)? Would you perform surgery (THA) again (yes/no)? Were your expectations of the operation met (no/light/moderate/strong/very strong)? How do you feel compared to before surgery (much better/better/same /worse/much worse)? Has your quality of life improved (no/light/moderate/strong/very strong)? How would you evaluate the function of your hip (normal/almost normal/unnormal/strongly unnormal)?

### Statistical methods

In advance, a power calculation was performed (based on the retrospective results of the first 100 ERAS patients of the department regarding the designated primary outcome variable “timed-up-and-go test”, *p* < 0.05, a priori sample size calculation required 100 patients per group to achieve a power of 0.8 with an expected drop-out-rate of 5%).

Continuous data are presented as mean (SD) or as median (IQR), depending on the distribution of the variables. Categorical variables are presented as absolute and relative frequencies. Comparisons between both treatment groups were performed by using either Student’s *t* test or the Wilcoxon–Mann–Whitney Test for continuous data or the Chi-square test of independence for categorical data. A *p* value < 0.050 was considered statistically significant for all tests. No impairment methods were used. All analyses were performed using Statistical Package for the Social Sciences (IBM SPSS Statistics for Windows, Version 27.0, IBM Corp., Armonk, NY, USA) and R (v4.2.1; R Core Team 2021).

## Results

### Patient characteristics

A total of 194 patients who underwent THA (98 ERAS/96 Conventional) were evaluated. Mean age was 64.31 (± 9.87) for ERAS and 65.55 (± 8.45) for the conventional group (*p* = 0.0582). Pre-surgery, there were no significant differences (*p* > 0.050) between the groups. No complications such as thromboembolic complications, fractures or revisions were recorded within the first week postoperatively in either study group.

### Mobility and range of motion (ROM)

**Fig. 2:**
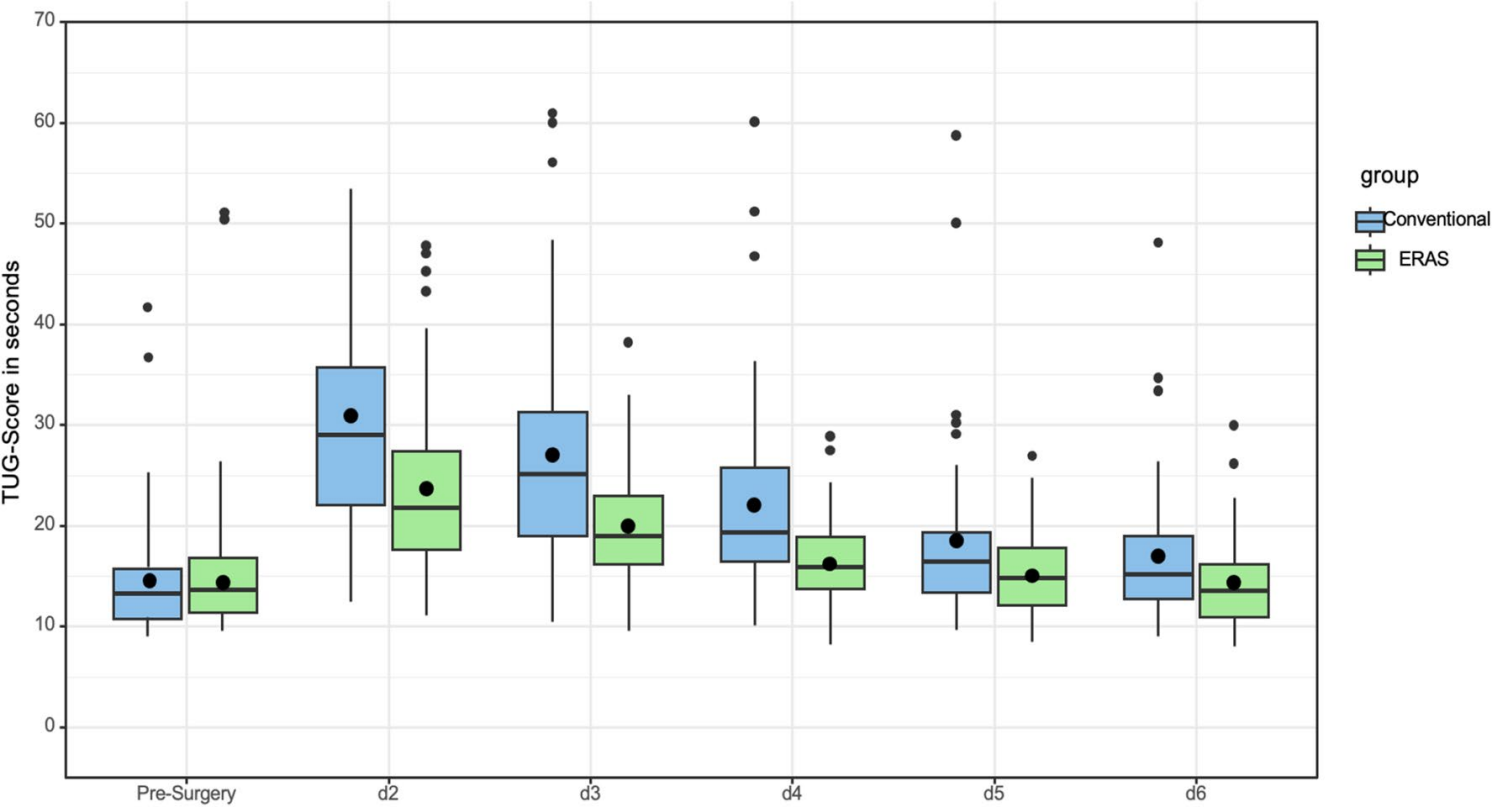
“Timed Up and Go Test” (TUG) pre-surgery and daily since day 2 up to day six after THA. All postoperative comparisons showed significantly better results in the ERAS group. The postoperative days are abbreviated as d1/d2 and so on

Before surgery, no significant differences between both groups regarding TUG, floor count and walking distance were detected. The ERAS group showed significant (*p* < 0.050) better TUG and walking distance results after surgery up to the sixth postoperative day compared to the conventional group (Table 1). The box plot diagram (Fig. 2) below shows the TUG results, as primary endpoint, of ERAS compared to the conventional group. ERAS group achieved significant better (*p* < 0.050) floor counts up to the third postoperative day compared to the conventional group (Table 1).

**Table 1:** Mean value and standard deviation of time up and go test (TUG) in seconds, floor count and walking distance in meter pre-surgery and daily starting 2 days after THA

Mobilization	ERAS	Conventional	*p* value
Pre-surgery			
TUG (sec)	14.11 (SD 4.43)	14.56 (SD 5.51)	0.567
Floor count	1.88 (SD 1.15)	1.74 (SD 1.2)	0.387
Walking distance (m)	1000 (IQR 500–2000)	625 (IQR 450–1000)	0.182
Day 2			
TUG (sec)	23.62 (SD 8.19)	30.91 (SD 13.5)	** < 0.001**
Floor count	0.32 (SD 0.67)	0.03 (SD 0.23)	** < 0.001**
Walking distance (m)	200 (IQR 100–306)	50 (IQR 30–138)	** < 0.001**
Day 3			
TUG (sec)	19.96 (SD 6.01)	27 (SD 11.14)	** < 0.001**
Floor count	0.76 (SD 0.83)	0.36 (SD 0.81)	**0.001**
Walking distance (m)	312 (IQR 236–500)	100 (IQR 70–300)	** < 0.001**
Day 4			
TUG (sec)	16.19 (SD 4.02)	22.02 (SD 9.03)	** < 0.001**
Floor count	1.51 (SD 1)	0.98 (SD 1.09)	**0.001**
Walking distance (m)	500 (IQR 400–800)	300 (IQR 179–600)	** < 0.001**
Day 5			
TUG (sec)	15.01 (SD 3.94)	18.49 (SD 8.86)	**0.006**
Floor count	1.39 (SD 1.01)	1.07 (SD 1.05)	0.037
Walking distance (m)	800 (IQR 500–1000)	500 (IQR 250–800)	** < 0.001**
Day 6			
TUG (sec)	14.36 (SD 4.44)	16.98 (SD 6.95)	**0.017**
Floor count	1.29 (SD 1.19)	1.26 (SD 1.13)	0.852
Walking distance (m)	1000 (IQR 550–1000)	600 (IQR 375–1000)	**0.007**

The *p* value of time up and go test (TUG) and floor count was calculated using the *t* Test. The *p* value of walking distance was calculated using the Wilcoxon-Test. The postoperative days are abbreviated as Day 1/Day 2 and so on. Statistically significant results are indicated in bold

**Fig. 3:**
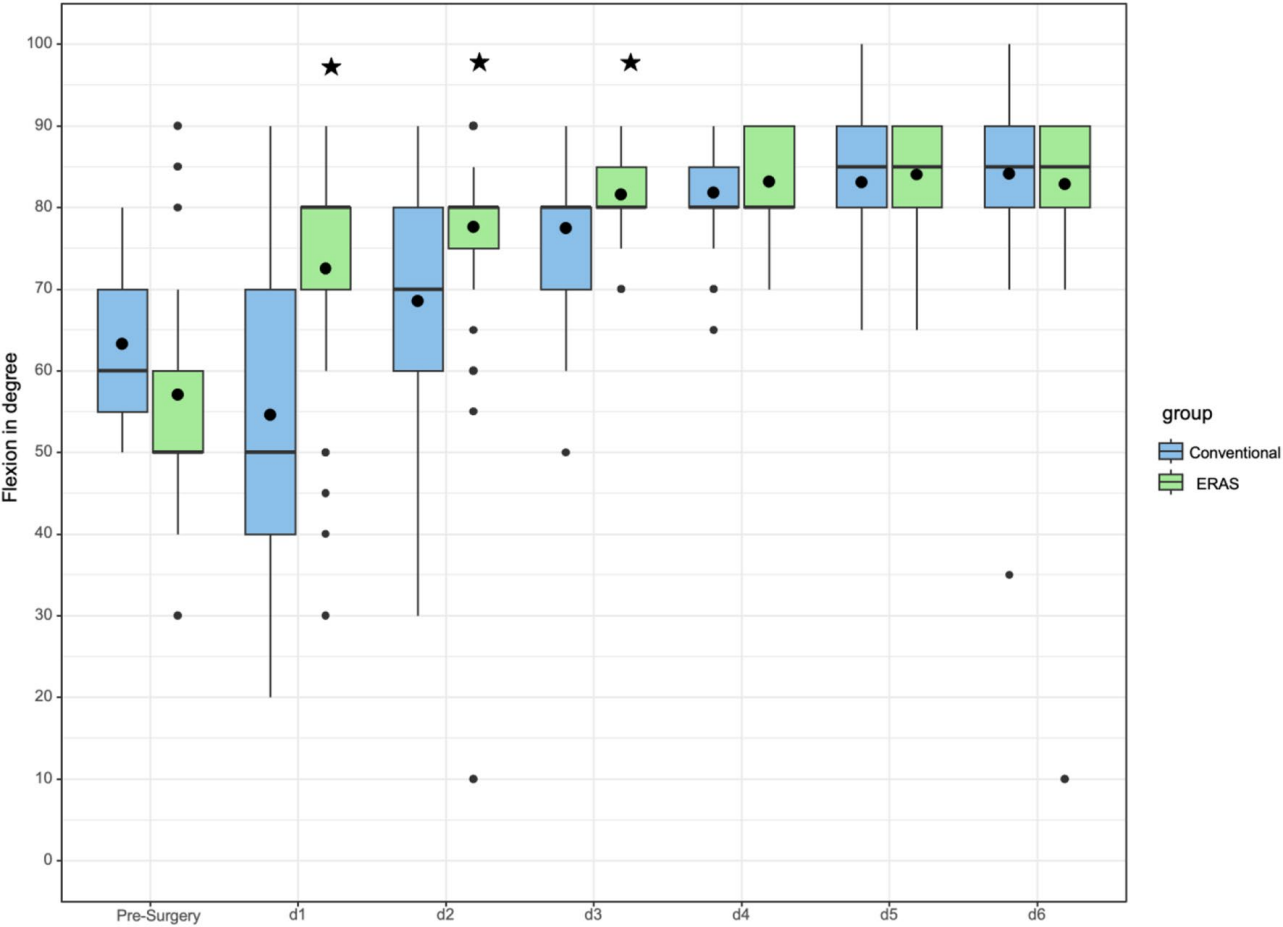
Flexion in degree of the operated hip pre-surgery and daily up to day six after THA. The stars indicate significant differences between the ERAS and the conventional group. The postoperative days are abbreviated as d1/d2 and so on

In addition to patient mobility, passive range of motion was also recorded daily by the independent physiotherapist. The Box-Plot diagram shows the results of passive hip flexion (Fig. 3) of ERAS and conventional patients preoperative and daily from the first to the sixth day after surgery. The Box-Plot diagram illustrates a significant better hip flexion (*p* < 0.001) of ERAS patients on the first postoperative day. Furthermore, on the third postoperative day ERAS patients showed a significant better hip flexion (*p* < 0.001) and abduction (*p* = 0.017) compared to the conventional patients. On the remaining days, we detected no significant difference regarding range of motion.

### Activities

Table 2 lists all postoperative *p* values of the subitems: getting up, personal hygiene, going to the toilet, dressing, sitting, walking. The p-values of ERAS compared to conventional group are calculated with the Wilcoxon test. On the first and second postoperative day, there were significant differences (*p* < 0.001) between ERAS and the conventional pathway in all subitems: getting up, personal hygiene, going to the toilet, dressing, sitting, walking. Except for personal hygiene, there were significant differences (*p* < 0.001) between ERAS and the conventional pathway until the third postoperative day. On the following days, no significant difference could be found.

**Table 2:** *p* Value of patient’s independent daily activities of ERAS group compared to the conventional group

Activities	Pre-surgery	Day 1	Day 2	Day 3	Day 4	Day 5
Getting up	*p* = 0.170	***p* < 0.001**	***p* < 0.001**	***p* = 0.005**	*p* = 0.638	*p* = 0.273
Personal hygiene	*p* = 0.167	***p* < 0.001**	***p* < 0.001**	***p* = 0.046**	*p* = 0.914	*p* = 0.300
Going to the toilet	*p* = 0.951	***p* < 0.001**	***p* < 0.001**	***p* = 0.001**	*p* = 0.252	*p* = 0.503
Dressing	*p* = 0.963	***p* < 0.001**	***p* < 0.001**	***p* = 0.001**	*p* = 0.073	*p* = 0.146
Sitting	*p* = 0.170	***p* < 0.001**	***p* < 0.001**	***p* = 0.009**	*p* = 0.154	*p* = 0.945
Walking	*p* = 0.974	***p* < 0.001**	***p* < 0.001**	***p* < 0.001**	*p* = 0.357	*p* = 0.927

The *p* value was calculated using the Wilcoxon test. The postoperative days are abbreviated as Day 1/Day 2 and so on. Statistically significant results are indicated in bold

### Pain

No significant difference between ERAS and conventional pathway was observed regarding postoperative pain on NRS (0–10). As shown in Table 3, decreasing values were obtained the following postoperative days with no significant difference between the groups.

**Table 3:** Mean value and standard deviation (SD) of pain measured with the NRS (Numerical analog scale): pre-surgery and daily starting on surgery day to day 5 after THA

NRS	ERAS	Conventional	*p* value
Pre-surgery			
Rest	4.39 (SD 2.42)	4.6 (SD 2.42)	*p* = 0.577
Mobilization	6.01 (SD 1.9)	6.2 (SD 1.65)	*p* = 0.485
Night	4.23 (SD 2.55)	5.37 (SD 2.36)	***p* = 0.002**
Surgery-day			
Rest	3.25 (SD 2.44)	4.15 (SD 2.3)	*p* = 0.058
Mobilization	3.65 (SD 2.32)	3.78 (SD 2.22)	*p* = 0.747
Night	3.73 (SD 2.35)	4.06 (SD 2.19)	*p* = 0.333
Day 1			
Rest	2.87 (SD 2)	2.67 (SD 1.79)	*p* = 0.467
Mobilization	3.66 (SD 1.7)	3.36 (SD 1.75)	*p* = 0.229
Night	2.9 (SD 2.15)	2.61 (SD 2.05)	*p* = 0.358
Day 2			
Rest	2.4 (SD 1.92)	1.92 (SD 1.63)	*p* = 0.072
Mobilization	2.97 (SD 1.82)	2.52 (SD 1.44)	*p* = 0.065
Night	2.21 (SD 1.92)	1.95 (SD 1.47)	*p* = 0.320
Day 3			
Rest	1.72 (SD 1.61)	1.64 (SD 1.35)	*p* = 0.741
Mobilization	2.17 (SD 1.52)	2.24 (SD 1.55)	*p* = 0.770
Night	1.88 (SD 1.67)	1.93 (SD 1.56)	*p* = 0.854
Day 4			
Rest	1.45 (SD 1.36)	1.56 (SD 1.4)	*p* = 0.610
Mobilization	1.9 (SD 1.27)	2.01 (SD 1.47)	*p* = 0.592
Night	1.6 (SD 1.49)	1.62 (SD 1.56)	*p* = 0.943
Day 5			
Rest	1.28 (SD 1.32)	1.5 (SD 1.28)	*p* = 0.281
Mobilization	1.55 (SD 1.21)	1.73 (SD 1.29)	*p* = 0.329
Night	1.38 (SD 1.55)	1.59 (SD 1.41)	*p* = 0.377

The *p* value was calculated using the *t* Test. The postoperative days are abbreviated as Day 1/Day 2 and so on. Statistically significant results are indicated in bold

### Patient assessment of the perioperative phase (PPP33)

Table 4 shows all PPP33 mean values, standard deviations and *p* values calculated using the *t* test. Altogether there were no significant differences between both groups.

**Table 4:** Mean value and standard deviation (SD) of PPP33 (Patient assessment in the perioperative phase) after THA

PPP33	ERAS	Conventional	*p* value
Total value	69.65 (SD 8.58)	69.78 (SD 6.39)	0.906
Information	24.21 (SD 2.7)	24.08 (SD 2.83)	0.757
Patient autonomy	15.01 (SD 2.39)	15.13 (SD 2)	0.717
Communication	10.18 (SD 2.71)	10.31 (SD 2.07)	0.720
Physical discomfort	11.02 (SD 2.3)	10.62 (SD 2.19)	0.237
Pain	10.18 (SD 1.46)	10.12 (SD 1.4)	0.763
Rest/recovery	7.51 (SD 0.89)	7.56 (SD 0.9)	0.723
Anxiety	7.29 (SD 1.06)	7.45 (SD 0.94)	0.270
Clinic performance	6.81 (SD 1.24)	7.02 (SD 1.18)	0.236

The *p* value was calculated using the *t* Test

*SD* standard deviation

### Patient-reported outcome measures (PROMs)

The PROMs were assessed 1 week after surgery. Patients of both groups stated that the surgery was successful in their eyes. Overall, no significant differences were found between both groups regarding the PROMs (Table 5).

**Table 5:** PROMs one week after surgery with ERAS or conventional setup

PROM	ERAS	Conventional	*p* value
Was the operation successful in your eyes?	Yes = 98%	Yes = 99%	0.540
	No = 2%	No = 1%	
Would you perform the surgery (THA) again?	Yes = 99%	Yes = 99%	0.980
	No = 1%	No = 1%	
Were your expectations of the operation met?	Very strong = 30%	Very strong = 30%	0.836
	Strong = 48%	Strong = 44%	
	Moderate = 16%	Moderate = 22%	
	Light = 5%	Light = 4%	
	No = 1%	No = 0%	
How do you feel compared to before surgery?	Much better = 50%	Much better = 40%	0.152
	Better = 42%	Better = 46%	
	Equal = 6%	Equal = 8%	
	Worse = 2%	Worse = 6%	
Has your quality of life improved?	Very strong = 19%	Very strong = 19%	0.525
	Strong = 39%	Strong = 27%	
	Moderate = 19%	Moderate = 32%	
	Light = 20%	Light = 20%	
	No = 3%	No = 2%	
How would you evaluate the function of your knee	Normal = 14%	Normal = 19%	0.325
	Almost normal = 74%	Almost normal = 72%	
	Impaired = 12%	Impaired = 9%	

The *p* value was calculated using the Wilcoxon test

## Discussion

This is the RCT clinical study regarding ERAS after THA over a period of 1 week in comparison to a conventional setup. The most important findings of the study were a significant better TUG (*p* < 0.05, primary outcome variable) and walking distance with comparable pain in the ERAS group compared to the conventional group during the first postoperative week. We demonstrated an excellent functional and subjective outcome. Moreover, the ERAS concept resulted in both a gain of patient’s independence and better daily activity during the first postoperative week. Our ERAS program has been achieved by focusing on a multidisciplinary collaboration and establishing ERAS units, with a well-defined organizational setup tailored to deliver an accelerated perioperative course of ERAS THA procedures.

### Mobilization

The TUG test has been used in many relevant studies in hip arthroplasty [17, 21], but in the literature, there was no comparable ERAS study found regarding TUG, walking distance or floor count after ERAS. After ERAS THA, we were able to reach a significant (*p* < 0.050) better mobility (TUG test) and independence including getting up, personal hygiene, going to the toilet, dressing, sitting and walking during the first 3 days. In ERAS pathways, early mobilization is considered as a cornerstone [22]. Wainwright et al. recommended the soonest possible mobilization of ERAS patients after surgery in a consensus statement [4]. Early mobilization must always be accompanied by good balance to decrease fall risk of senior citizens [24]. After THA, 23.2% people fell during the first 12 months after surgery [23]. A valid tool for screening balance deficits is the TUG test [24]. With the help of this simple mobility test, the mobility or body balance can be assessed and the resulting risk of a fall, especially of the ageing person, can be determined. As analyzed by univariate regression analysis, the TUG test was significantly associated with LOS, too [25]. We could prove that early mobilization leads to a significant (*p* < 0.050) better TUG test in the postoperative course. When patients are discharged, they must be able to walk a distance safely and climb stairs (discharge criteria). Another advantage of mobilization and walking on the operation day after THA was the independence and improved quality of life [26]. We recognized significantly (*p* < 0.050) better results in the ERAS group regarding ROM, walking distance and the floor count. As soon as the second postoperative day, ERAS patients were able to walk 252.77 m whereas conventional patients were able to walk 119.92 m (*p* < 0.050). Previous studies have already demonstrated the importance of accelerated physiotherapeutic protocols. Hereby, favorable results in gait and muscle strength were possible [27]. Intensified active treatment and additional mobilization which started on the operation day after joint replacement achieved enhanced results. Matheis et al. recorded significant higher ROM (flexion *p* < 0.050, abduction *p* < 0.050) and less muscle atrophy [28]. In contrast to that, Chen et al. showed no significant difference in terms of functional capacity and muscle strength recovery compared to standard rehabilitation [29]. Overall, ERAS is not just increased physiotherapy. ERAS is a multidisciplinary setup which achieved significant better results compared to conventional treatment after THA.

### Pain and complications

Patients are usually allowed full weight bearing immediately after THA, but this can increase postoperative pain and consequently affect opioid consumption [30]. Therefore, multimodal pain management seems to be a gamechanger in the operative care of patients undergoing THA. Meticulous evaluation and management of pain was pivotal in ERAS for the avoidance of postoperative complications and decreased mobility [31]. Sufficient pain management included intraoperative anesthesia, too. A network meta-analysis conducted in 2018 showed that the most superior outcomes after THA relating to pain management were achieved with spinal anesthesia in the first 24 h [32]. The interdisciplinary process optimization of all treatment pathways led not only to better mobilization but also to decreased pain scores. Although ERAS patients had more physical therapy and intensive mobilization training, we have observed that ERAS patients had comparable pain levels at rest, at mobilization and at night to patients of the conventional pathway during the complete observation period.

Like Scott et al., we were able to verify the lack of complications during the observation period, too [43]. In contrast, Li et al. demonstrated a higher overall complications rate regarding ERAS in a same-day discharge setting in comparison to an inpatient setting [33]. Ripollés-Melchor et al. described that in high-risk patients (ASA scores of III–IV) ERAS THA did not result in better outcomes, although there were fewer readmissions [34]. In general, ERAS programs in THA have been proven safe and beneficial regarding complication and readmission rate [5].

### PPP33

A fundamental characteristic of the quality of healthcare is patient satisfaction, defined by the WHO [35]. Many international published studies have used scores like WOMAC, EQ-5 D, SF36, and Harris Hip Score (HHS) to evaluate patient satisfaction and quality of life in patients after ERAS THA [15, 36–38]. These studies confirm a significantly (*p* < 0.010) better quality of life and functional outcome. However, patient satisfaction perioperatively was only a minor subitem in the questionnaires. So far, no study had investigated the acceptability of ERAS THA to patients through a targeted questionnaire. The PPP33 questionnaire assesses patient satisfaction and patient acceptance of surgical procedures during the perioperative period [20]. Not only the randomized study design but also the PPP33 score were to be emphasized. We could show good overall PPP33 score for ERAS and the conventional THA (*p* = 0.906). Furthermore, the subitems information (*p* = 0.757), patient autonomy (*p* = 0.717), communication (*p* = 0.720), physical discomfort (*p* = 0.237), pain (*p* = 0.763), rest/recovery (*p* = 0.723), anxiety (*p* = 0.270), and clinic performance (*p* = 0.236) showed satisfying results in both groups. We could not prove that patients were more satisfied after ERAS THA than after conventional THA. This is being interpreted that surgeons have reached such a high postoperative satisfaction standard after THA, that patients are generally very satisfied, independent whether a conventional or ERAS setup is applied.

It was not proven that the results of this validation equally apply to our orthopedic study collective but the questionnaire has already been used in an internationally published study of elective surgery with general anesthesia [39]. For this reason, it is the first time that not only the postoperative phase but also the perioperative phase was assessed after ERAS surgery.

### PROMs

In addition to the functional mobility, range of motion, pain and PPP33 questionnaire we added subjective patient-reported outcome measures (PROMs—Table 5). Prodinger et al. succeeded in integrating PROMs into health information systems on a national scale [40]. PROMs evaluate the expectations and perspective of the patient [41]. Consequently, PROMs matter more to patients than the impact on cost saving or length of hospital stay. Measuring PROMs after THA could be a key tool to improve healthcare quality. By asking the questions: “How do you feel compared to before surgery? Was the operation successful in your eyes? Has your quality of life improved? Would you perform the surgery (THA) again? Were the expectations of the operation met?” we wanted to evaluate if the patient’s expectations were fulfilled in the early postoperative course after 1 week. Fortunately, both groups were very satisfied with the surgery and postoperative outcome, with no significant difference. Almost 100% of the study patients rated the ERAS THA as successful and that they would perform ERAS again. In addition, 91% ERAS patients felt “much better” or “better” in the early postoperative course compared to preoperatively. In conclusion ERAS resulted both in excellent functional outcome and a high level of patient satisfaction.

### Limitations

The present study was conducted in a single-blinded study design due to regulations in Germany (medication used intraoperatively only in the ERAS group), therefore, the surgeon had to know which study group patients were assigned to. Nevertheless, data were evaluated by an independent team of physician assistants and physiotherapists, therefore, bias should be reduced to an absolute minimum. Secondly, immobilized patients were excluded in this study, leaving unclear how ERAS THA affect the success of an older immobile and patient collective with comorbidities. To guarantee standardized and controlled study conditions, the follow-up period in our study was limited to one week, so mid-/long-term results are not available. Nonetheless, the short follow-up could also be stated as a strength of the study as the clear and standardized setup with two arms and equal LOS considering THA can rarely be found within other countries’ health care systems. With this elaborate prospective randomized study, qualitative conclusions about postoperative outcome can be made. Most high-quality studies on this topic were surveying length-of-stay (LOS), but to date, no data was available regarding mobilization, function, pain and satisfaction—the reason, why we conducted this study. In the future, prospective multi-center longer term studies in a randomized controlled study design should be undertaken to extend the acquired knowledge in this study and measure and conclude pain, functional outcome and quality of life improvements after ERAS THA.

## Conclusion

This prospective single-blinded randomized controlled clinical study substantiated that ERAS concepts are effective regarding clinical outcome and can be used in daily clinical practice. ERAS patients after cementless THA showed significant better TUG scores, daily mobility and a high patient satisfaction, while the pain level was not inferior regarding the conventional pathway. During a follow-up period of 1 week, pain scores decreased and function scores increased to excellent values. PPP33 and PROMs confirmed patient satisfaction and acceptance of the ERAS surgical procedure. Due to the results, all patients are now treated with the ERAS protocol in our department, including fragile and multimorbid patients. Seeing the non-inferiority considering pain and patient satisfaction as well as results proving superiority considering mobility and patient independence, the next step should be fine-tuning of the multiple components in ERAS THA to enable patients with high co-morbidity burden or special needs a safe and effective ERAS THA—“ERAS for every patient but with different targets”.

## Data Availability

The availability of data and materials is detailed described in the methods.
